# Physicians’ attitudes to disability pension – impact of diagnosis: an experimental study

**DOI:** 10.1186/s12913-020-06043-2

**Published:** 2021-02-05

**Authors:** Ashley McAllister, Allison Milner, Monika Engblom, Patrick Corrigan, Bo Burström

**Affiliations:** 1 Department of Global Public Health, Karolinska Institutet, Stockholm, Sweden; 2 Melbourne School of Population and Global Health, The University of Melbourne, Melbourne, Australia; 3 Department of Neurobiology, Care Sciences and Society, Karolinska Institutet, Stockholm, Sweden; 4 Department of Psychology, Illinois Institute of Technology, Chicago, USA

**Keywords:** Alcohol: depression: diagnosis, Disability income support, Mental health: stigma, Sweden

## Abstract

**Background:**

The purpose of this study is to increase understanding of physicians’ attitudes towards disability pension applicants, and the impact of diagnosis. We hypothesize that physicians are more likely to think that patients with physical illnesses should get a disability pension than those with mental illness or alcohol dependence. Disability pension is an important source of income for those unable to work because of a disability and type of diagnosis should not impact accessing these benefits.

**Methods:**

We conducted an experiment with a 2 by 3 factorial structure in Sweden. Each physician was randomly assigned one of six patient vignettes, with the same background description but with a different diagnosis. Each vignette had a diagnosis of either depression, alcohol dependence or low back pain, and was about a man or a woman. Logistic regression was used to examine the odds of a physician reporting that a patient should get a disability pension. Effects are reported in terms of odds ratios (ORs).

**Results:**

1414 Swedish registered physicians in psychiatry or general practice (24% response rate) completed the survey. Physicians assigned the alcohol dependent vignette had OR 0.45 (95% CI: 0.34 to 0.60) for perceiving that a patient should get a disability pension compared to physicians assigned the low back pain vignette. Physicians assigned the depression vignette had OR 1.89 (95% CI: 1.42 to 2.50) for perceiving that a patient should get a disability pension compared to physicians assigned the low back pain vignette.

**Conclusion:**

The patient diagnosis was associated with the physicians’ response regarding if the patient should get a disability pension. A physician’s perception is likely to impact a patient’s access to disability pension.

**Supplementary Information:**

The online version contains supplementary material available at 10.1186/s12913-020-06043-2.

## Background

In Sweden, long-term sickness absence benefits and disability pension provide financial security for those that are unable to work as a result of a long-term disability. In 2017, approximately 350,000 persons were receiving disability pension and half of new recipients had a mental disorder [1]. The Swedish Government tightened the eligibility criteria in 2003 and 2008 and set a maximum time for being on sickness absence making it much more difficult to access these benefits [1]. The impact of disability pension rejection greatly depends on a person’s circumstances. Persons not considered eligible for sickness absence benefits or disability pension are expected to work or seek work. The last resort, if work is not possible, is to apply for means-tested income support (social assistance), which is managed by the municipal social services at a much lower level. Persons with mental illnesses are more likely to be in more economically vulnerable positions than persons with other diagnoses [2]. To be eligible for the disability pension, a person must be between the ages of 30–64 years and have a permanently reduced work capacity of at least one quarter because of a medical disability. For those under 30 years, access to disability pension is only temporary (in most cases for 3 years before re-application).

### The role of diagnosis in disability pension

In the disability pension process, a diagnosis is important as it establishes a medical reason for reduced work capacity. However, it is unclear in the literature whether the *type* of diagnosis may matter in this process. Some evidence suggests a hierarchy of disability exists [3–5], meaning some disabilities are valued as more deserving than other disabilities. Most studies conclude that physical disabilities are at the top of this hierarchy, followed by mental illnesses then alcohol, and other dependences at the bottom [6, 7]. Rationale for this hierarchy is that persons with a physical disability are perceived as less blameworthy, the (bio-)medical nature of the illness (i.e. there are diagnostic tests available), and lower/no stigma attached to the disability [3, 8] compared to many psychiatric disabilities. In this study, we examine alcohol dependence, recurrent depression, and lower back pain with sciatica (hereto referred to as low back pain). These diagnoses represent a significant burden of disease [8–10] and are subject to clinical ambiguity [3]. Hatcher and Arroll [11] argue that such conditions constitute “a clinically, conceptually and emotionally difficult area” making them interesting conditions to explore whether the diagnosis matters in the disability pension process.

### The role of gender in disability pension

In theory, gender should have no bearing on whether a person gets a disability pension. However, some evidence suggests that gender stereotyping exists in health care and the stereotypes discriminate against women [12–14]. One qualitative Norwegian study found that health care workers described female patients as demanding compared male patients [14]. An experimental study found that nurses were more likely to provide comprehensive treatment to men compared to women [15]. However, more knowledge is needed on what role do attitudes towards diagnosis *and* gender play (if any) in the disability pension process. In this study, we address these gaps.

### The role of the physician in the disability pension process

Disability pension was designed with physicians as neutral data gathers [16] meaning they are to provide objective information about their patients without their opinions influencing the process. However, in reality, the medical assessment process is complicated. In Sweden, the physician is an informal gatekeeper in this process. Physicians do not make any eligibility decisions but the evidence they provide in the medical assessment required to substantiate a disability pension application is pivotal. Furthermore, we know that physicians, similar to the general population, hold similar negative attitudes towards people with mental illnesses [17–21] And that these negative attitudes can contribute to inequalities in healthcare [18] including diagnostic overshadowing [19, 21]. To the best of our knowledge, physicians’ attitudes towards patients with mental illness has not been explored in the Swedish context. As such, we wanted to better understand how physicians’ negative attitudes might impact the disability pension process in Sweden.

### Aims of the study

We aim to increase understanding of physicians’ attitudes towards disability pension applicants with mental illness and alcohol dependence compared to low back pain. We expect that the hierarchy of disability will translate into the disability pension setting. As such, we hypothesize that physicians’ perception of whether a patient should get a disability pension is *dependent* on whether the patient has a mental or physical illness. Specifically, we hypothesize that perceptions about whether a person should get a disability pension will be less for alcohol dependence and depression compared to low back pain. We also hypothesize that perceptions regarding getting a disability pension may be more negative for the female vignette compared to the male vignette patient.

## Methods

### Study design

We used Rossi and Nock [22]‘s experimental design, varying the diagnosis and gender of the vignette patient (3 × 2 factorial design). The literature shows that “vignettes are a valid, reliable, inexpensive, and practical method for assessing clinical practice” [23]. To test our hypothesis, the vignettes were identical in information except for patient name (Johan for the man; Johanna for the woman) and the diagnosis. Each vignette contained information about year of diagnosis, symptoms, current treatment, and brief work history. Each vignette person met the basic criteria for disability pension in Sweden and therefore each vignette patient had a theoretically equal chance of getting a disability pension. The vignettes were largely created through the use of an expert panel (see below for more detail). This study was approved by the Stockholm Regional Ethics Board (Dnr 2018/683–31/5).

#### The vignettes

We created six vignettes illustrating both sexes and three different diagnoses – alcohol dependence, depression, and low back pain. These diagnoses represent: common conditions for receiving disability pension, ‘subjective’ diagnoses – mostly invisible and difficult to prove with diagnostic tests and varying levels of moral status attached to the conditions. Except for specific information about the particular diagnosis and sex of the patient, we kept all other information constant, for example:

- The patient is 47-years-old and diagnosed 10 years ago
- Exhausted all treatment options – medications and therapy (with a psychologist)
- On and off sick-leave for 5 years and 100% sick leave in the past 12 months
- Unable to work more than nine hours per week in previous years and
- Occupational therapist reports that the patient is difficult

Most of this information correlates with information that is requested by the Swedish Social Insurance Agency to determine eligibility such as:

- Must be over 30 years of age
- Permanent condition (or likely to last long)
- Exhausted all treatment and rehabilitation options
- Previously on sick-leave and now unable to work more than 10 h per week

As such, the vignettes meet the minimum requirement for disability pension and theoretically could be deemed eligible. We aimed to design these vignette persons in the ‘grey zone,’ meaning physicians should find it difficult to immediately determine whether disability pension is the most appropriate option for the vignette person. Furthermore, given the health conditions are subjective with little objective evidence to prove their condition, physicians’ attitudes may be more likely to influence decisions.

We intentionally omitted personal details about the patient (i.e. educational level, specific job title, living situation (e.g. single parent with two children)) since technically physicians should not consider such factors during the Swedish disability pension medical assessment.

To ensure that vignettes were similar enough to compare, we described symptoms that were present in all three conditions such as fatigue, difficulty maintaining relationships, and difficulty concentrating (see Supplementary File 1 for example vignette).

We presented the vignettes as “paper-people” to impose lower cognitive demands and less time than video vignettes [24]. We recognise that a limitation of this choice is that the physicians are unable to see cues or gauge for themselves the level of severity of impairment of the fictitious patient. However, disability pension eligibility criteria remove the context of the patient to focus on objective medical ‘facts’ so the written vignettes provided the best platform to achieve this goal.

### Expert consensus panel

In this study, we engaged an expert panel comprised of physicians (psychiatrists and general practitioners), psychologists, researchers, and representatives from the Swedish Social Insurance Agency, all working in the area of disability pension (or providing help to their patients). The expert panel contributed through developing the patient vignettes (including consensus on diagnosis, treatment, and previous sick-leave), questionnaire items, and general advice through the project. For example, the panel suggested that gender of the patient was important and should be an additional exposure variable and aligns with the literature on mental illness, stigma, and gender [25]. We achieved consensus among the expert panel about the diagnosis, gender and context. As such, the expert panel’s input contributed to making sure the vignettes were realistic and increasing the overall external validity of the study.

### Setting

The survey was conducted in Sweden. Statistics Sweden administered the vignette questionnaire in October 2018. Physicians had approximately 6 weeks to answer the questionnaire either online or via a paper version in a place convenient to them. Reminders were sent at two and 4 weeks.

### Participants

Eligibility criteria were (i) a registered Swedish physician (ii) specialising in general practice (GP) or psychiatry. We sampled GPs and psychiatrists because these are the two groups of physicians that primarily complete a medical assessment for disability pension in Sweden. It should be noted that in Sweden, general practice is a speciality and requires additional training (about 5 years). Physician addresses were received from Hälso och sjukvårdens adressregister. We sent the questionnaire to all registered psychiatrists (*n*=1783) and randomly selected GPs (representing approx. two thirds of total GPs in Sweden) (*n* =4217). A total of 1414 persons responded to the survey (23.6% response rate). Reason for non-response was not collected.

### Data sources and measurement

Data came from two sources: register information from Hälso och sjukvårdens adressregister (physicians’ gender, age, and location) and self-reported information through a questionnaire. Each participant was randomly assigned one of the six vignettes, but the questionnaire remained the same regardless of the assigned vignette. The questionnaire pertained to the hypothetical patient in each vignette. The questionnaire had two sections. The first included questions related to disability pension such as “Do you think this patient should get a disability pension?” or “Do you think it is likely to that this patient has exaggerated their symptoms. The second used the highly validated Attribution Questionnaire to measure stigma. In this study, we focus on reporting results from the first section of the questionnaire. In this section, physicians were asked to rate how much they agree with each statement made about the hypothetical patient on a Likert scale from 1 to 4. Cognitive testing (*n*=3) and pilot testing (*n*=20) was conducted to test for internal and external validity. We amended the questionnaire based on feedback provided during these tests.

#### Variables of interest

The outcome of interest was whether a physician perceives that the patient should get a disability pension. We measured this by asking “Do you think this patient should get a disability pension?” (hereto referred to as ‘should get a disability pension’). There were four response categories (i) definitely not (ii) probably not (iii) probably yes (iv) definitely yes. Physicians also had the option of choosing ‘don’t know’ but as this category was small (94, 6.7%) we excluded it in analysis. For purposes of analysis, we dichotomised the question combining responses (i) and (ii) to represent ‘no’ and (iii) and (iv) to represent ‘yes’.

We have two independent variables – patient diagnosis and patient gender. Patient diagnosis is 3-categories: (i) recurrent depression (ii) alcohol dependence and (iii) low back pain. Patient gender is binary: (i) men (ii) women. We had six vignettes in total, representing three diagnoses and two genders. Hälso och sjukvårdens adressregister randomly assigned each physician into the six strata. Each stratum was one third psychiatrists and two third GPs.

#### Covariates

Based on previous literature [26, 27], we identified physician age, gender, and experience in disability pension as potential variables likely to influence perception of should get a disability pension. Age was measured continuously, gender was binary (men/women), and experience was measured by number of disability pension assessments completed in the past 12 months (none; 1–4; 5–10; more than 10). We also recognize that physician speciality could influence the outcome measure but we did not have this data so were unable to include speciality as a confounder. However, we know at an aggregate level how many were in the sample: 443 psychiatrists and 971 GPs.

### Statistical methods

We calculated descriptive statistics for all variables of interest. Following this, we conducted binary logistic regression to examine the main hypothesis. We tested for main effects of patient diagnosis and patient gender, and the interaction between gender and diagnosis. Estimates presented include those from the adjusted models include the covariates. We found no statistically significant results for interaction. As such, we only present the main effects (see Table 2) but provide interaction results in Supplementary File 2. Analysis was undertaken in Stata 15.1.

#### Sensitivity analysis

We compared the distribution of physician age, gender, location and type of physician of the invited sample (*n* = 6000) with the respondents (*n*=1414) and final analytic sample (*n*=1101) (see Supplementary File 3). The distribution remained relatively similar across all three.

## Results

### Descriptive data

Table 1 summarises the socio-demographic characteristics of the physicians. More than two thirds of physicians were GPs (68.7%). Slightly less women responded than men (51.1% men vs. 48.9% women). Few young physicians (less than 35 years old) responded and the average age of physicians was 53 years. 41% of physicians were located in one of the three major Swedish cities (Stockholm, Malmö or Göteborg). Over 40% of physicians reported they completed five or more disability pension assessments in the past 12 months. 12% of physicians said they completed none in the past 12 months. More than half (54%) of physicians said the vignette patient should get a disability pension; only 0.8% of physicians chose not to answer this question. The response rate for physicians with the alcohol dependent vignette (30.6%) was slightly lower compared to physicians with the depression or back pain vignette (35.0 and 34.4%, respectively).

**Table 1:** Socio-demographic characteristics of the sample (*n* = 1414)

Variable	Frequency (n)	Percentage (%)
Female physicians	691	48.9
Location of physician^a^		
Major city	580	41.0
Regional or rural	834	59.0
DP assessments completed last 12 months		
None	174	12.3
1–4	584	41.3
5–10	338	23.9
> 10	261	18.5
Missing	57	0.4
Do you think the patient should get a DP?		
Yes	765	54.1
No	543	37.8
Don’t know	94	6.6
Missing	12	0.8
Vignettes patients with		
Depression	495	35.0
Alcohol dependence	432	30.6
Low back pain	487	34.4

^a^Physician address provided could be either workplace or home address

Abbreviations: *DP* Disability pension

### Main results

Table 2 summarises the results from the logistic regression showing the crude and adjusted results. Physicians with the depression vignette had OR 1.89 for rating the patient as should get a disability pension compared to physicians with the low back pain vignette. This association increased to OR 1.93 after adjusting for covariates. Physicians with the alcohol dependent vignette were much less likely to report that the patient should get a disability pension compared to physicians with the low back-pain vignette (OR 0.45, 95% CI 0.34 to 0.60). This crude association increased after adjusting for covariates (OR 0.44, 95% CI 0.33 to 0.59).

**Table 2:** Effect of vignette diagnosis and gender on the odds of a physician thinking ‘should get a DP’

	Variable	Crude Model (***n***=1308)	Adjusted Model (***n***=1255)
		OR (95% CI)	OR (95% CI)^a^
Should get a DP	Vignette diagnosis		
	Low back pain	1	1
	Depression	1.89 (1.42 to 2.50)	1.93 (1.44 to 2.59)
	Alcohol dependence	0.45 (0.34 to 0.60)	0.44 (0.33 to 0.59)
	Vignette gender		
	Male	1	1
	Female	0.91 (0.72 to 1.14)	0.93 (0.74 to 1.18)

Abbreviations: *DP* Disability pension; *OR* Odds ratio; *CI* Confidence Interval

^a^Model adjusted for age of physician, gender of physician and experience of physician completing disability pension assessment in past 12 months

In terms of gender, the results show that physicians were slightly less likely to think the female vignette should get a disability pension compared to the male vignette but the results were not statistically significant (OR 0.91, 95% CI 0.72 to 1.14).

Table 3 summarises the results from the logistic regression showing the crude and adjusted results stratified by physician gender. Male physicians with the depression vignette had OR 1.38 for rating the patient as should get a disability pension compared to male physicians with the low back pain vignette. Male physicians with the alcohol dependent vignette were much less likely to report that the patient should get a disability pension compared to male physicians with the low back-pain vignette (OR 0.31, 95% CI 0.21 to 0.45). Female physicians with the depression vignette had OR 2.61 for rating the patient as should get a disability pension compared to physicians with the low back pain vignette. Similar to male physicians, female physicians with the alcohol dependent vignette were also much less likely to report that the patient should get a disability pension compared to female physicians with the low back-pain vignette (OR 0.67, 95% CI 0.45 to 0.99). This crude association slightly increased after adjusting for covariates (OR 0.62, 95% CI 0.41 to 0.93).

**Table 3:** Effect of vignette diagnosis and gender on the odds of a physician thinking ‘should get a DP’ by physician gender

	Variable	Male Physicians		Female Physicians	
		Crude Model (***n***=673)	Adjusted Model (***n***=650)	Crude Model (***n***=634)	Adjusted Model (***n***=605)
		OR (95%CI)	OR (95%CI)^a^	OR (95%CI)	OR (95%CI)^a^
Should get a DP	Vignette diagnosis				
	Low back pain	1	1	1	1
	Depression	1.38 (0.91 to 2.08)	1.37 (0.90 to 2.09)	2.61 (1.75 to 3.87)	2.64 (1.75 to 4.0)
	Alcohol dependence	0.31 (0.21 to 0.45)	0.31 (0.21 to 0.47)	0.67 (0.45 to 0.99)	0.62 (0.41 to 0.93)
	Vignette gender				
	Male	1	1	1	1
	Female	1.08 (0.76 to 1.49)	1.07 (0.76 to 1.45)	0.78 (0.56 to 1.08)	0.81 (0.58 to 1.13)

Abbreviations: *DP* Disability pension; *OR* Odds ratio; *CI* Confidence Interval

^a^Model adjusted for age of physician and experience of physician completing disability pension assessment in past 12 months

## Discussion

In this experimental study, we investigated the association between patient diagnosis and whether a physician thinks the patient should get a disability pension in Sweden. The results partially supported our hypothesis that physicians had a greater probability of thinking that patients with mental illness should not get a disability pension compared to patients with physical illness, with respect to the results for the alcohol dependent vignette. However, the magnitude of this effect was surprising given that all vignettes had a theoretically equal chance of getting a disability pension. One explanation could be that persons with alcohol dependence are at higher risk of stigma and structural discrimination because their diagnosis is often not considered a mental illness and attributed much more to personal responsibility [8]. More research is needed on structural discrimination and alcohol dependence to unpack potential stigma and discrimination.

Results for the depression vignette were opposite to what we hypothesised; physicians had greater odds of thinking that a patient should get a disability pension compared to the patient with low back pain. We found one other study where participants ranked depression slightly higher than sciatica (mean 4.3 and 4.2, respectively) in terms of disease prestige [3]. Similar to many mental illnesses and alcohol dependence, low back pain often lacks objective medical tests. As such, the pain is sometimes associated with “psychosomatic pain” [3]. Grue et al. [3] argue that somatisation can lead to perceptions of malingering. Future research should explore physicians’ perceptions of malingering, the role of diagnosis and how those perceptions might mediate whether the physician thinks the vignette patient should get a disability pension.

Overall, our results support that there could be a disease hierarchy that exists for disability pension in Sweden. Given that the three diagnoses examined are the most common reasons for applying for a disability pension in Sweden and many other countries, it is important to further examine why this hierarchy exists and to ensure the hierarchy does not lead to inequalities of access to disability pension. In recent years, a centralization of decisions on granting disability pensions in Sweden has resulted in an increase in the rate of rejection of applications from 50 to 70%. Hence an increasing number of persons unable to work because of poor health must instead resort to social assistance [28]. While our study is experimental, a Norwegian study using register data on *actual* rejected disability pension applications support our results. Galaasen et al. [2] show that applicants with drug and alcohol dependence or complex musculoskeletal are more likely to be rejected compared to other somatic conditions [2].

The female vignette patients were perceived slightly more negative than male vignette patients but the results were not statistically significant. It seems, in this study, that the diagnosis or hierarchy of disability is more important when it comes to perceptions about whether a patient should get a disability. However, more research particularly qualitative inquiry could provide more insight into what role gender of the patient might play in the disability pension process.

Our research suggests that different attitudes could exist between male and female physicians towards disability pension applicants. Our findings support the literature on gender differences towards people with mental illness [29, 30]. Overall, women in general (not physicians specifically) are more likely to behave kindlier [29, 30]. In the sickness insurance literature, female physicians are more likely to sick-list patients than male physicians regardless of the diagnosis [27]. However, a gap still exists in understanding why these differences exist. Future research, mainly qualitative research, should explore the role that physician gender might play in assessing disability pension in greater depth. A greater understanding of the role that physician gender might play could also provide more insight into what interventions could reduce disease hierarchy.

In most Organisation for Economic Co-operation and Development countries, people leaving the workforce due to illness and disability, especially for disability pension, is a problem. To the best of our knowledge, this is the first experimental study to examine physicians’ attitudes towards disability pension applicants in Sweden. In fact, studies seldom explore disability evaluation (the gateway to these programs) at all. One systematic review on inter-rater reliability found high variation among raters and urgently called for more research on disability evaluation [31]. Lax et al. [32] propose that differences in evaluation outcomes are based more on differences in opinion rather than skills or training. Our study builds on the findings by Lax et al. [32] by showing that the type of diagnosis can influence physician’s perceptions about whether a patient should get a disability pension. Unlike other studies that tend to include fewer (no more than 100) physicians (raters) but many health conditions [31], this current study focused on three conditions but included over a thousand physicians (raters).

### Limitations of the study

The response rate is only about a quarter of the invited study population and slightly lower than anticipated but well above our power calculation and desired sample of about 500 physicians. We expected a response rate of about 30% (compared to ours of about 24%) based on based on declining rates of previous Swedish physician surveys [33, 34]. Some of the demographics are skewed e.g. the average age of the physician is slightly older compared to other surveys of Swedish physicians [27, 34] (See Supplementary File 3). Our study is ultimately hypothetical and does not represent what the physicians *actually* do in practice, though some evidence supports that vignette studies are correlated with actual physician behavior [23]. We also cannot ignore that physicians were completing this survey in the context of Swedish disability pension with increasingly strict eligibility criteria. As such, physicians’ responses may to some extent reflect the prevailing policy climate in Sweden. Finally, although the treatments between the vignette patients were similar e.g. medication and psychological therapy, the pharmaceutical component varied across the three diagnoses. Through consensus, the expert panel and research team agreed the treatments were all comparable but this could have introduced additional bias into the experiment.

## Conclusion

In conclusion, our study highlights the complexity that exists in the disability pension process. Physicians will continue to play a pivotal role in disability assessments. However, too little training exists to help them with this process. In Sweden, some basic training is provided by the medical schools and there are some mandatory courses of insurance medicine for specialists (both GPs and psychiatrists). In Stockholm, the Academic Primary Care Centre for GPs provides “refreshment” courses but that could also be done elsewhere in Sweden. We recommend that medical educators consider including more training in disability pension medical assessments. Similar studies should be conducted in other contexts to see what role context plays in the findings presented. Physicians are essential in most disability pension processes, and it is crucial that despite its complexity, we reduce any inequalities that might exist in the disability pension process to ensure that those who need disability pension receive it.

## Supplementary Information

**Additional file 1: Supplementary File 1.** Example of Patient Vignette. **Supplementary File 2.** Interaction effect of vignette diagnosis and gender on the odds of a physician thinking ‘should get a DP’. **Supplementary File 3.** Characteristics of the initial study population, respondents and analytic sample.

## Data Availability

The data are not publicly available due to privacy or ethical restrictions.

## Abbreviations

CI: Confidence intervals
DP: Disability pension
GPs: General practitioners
ORs: Odd ratios
