# Supplementary material for: Physicians’ attitudes to disability pension – impact of diagnosis: an experimental study
Source: BMC Health Serv Res. 2021 Feb 5;21:122. doi: 10.1186/s12913-020-06043-2 (PMC7863496; doi:10.1186/s12913-020-06043-2)
Supplement: Supplementary file 1 — Additional file 1: Supplementary File 1. Example of Patient Vignette. Supplementary File 2. Interaction effect of vignette diagnosis and gender on the odds of a physician thinking ‘should get a DP’. Supplementary File 3. Characteristics of the initial study population, respondents and analytic sample. [file 12913_2020_6043_MOESM1_ESM.docx]

**Supplementary File 1. Example of Patient Vignette**

[Johan/Johanna] is 47 years and ten years ago he was diagnosed with [severe alcohol dependence (ICD-10 F10-.2)]. Since then, he has been admitted to hospital two times due to their alcohol dependence.

He has self-reported a strong craving for alcohol and also states that he drinks at work which makes it difficult to concentrate. During relapses, Johan says he has problems performing certain daily routines such as shopping and cleaning. He often comes late to work or is completely absent due to fatigue related to their alcohol dependence. Because of Johan’s illness, he often ends up in conflicts with his managers and colleagues. Even in periods of sobriety, he states that he still has some of these symptoms. At his most recent workplace, an occupational therapist reported from Occupational Health Care that he has observed these symptoms, and that Johan can be demanding, rude and sometimes aggressive.

Johan has been prescribed Antabus to reduce his alcohol cravings, but he drinks alcohol despite taking the medicine. He has participated in a support group at an addiction centre led by a psychologist. He has been to rehabilitation for alcohol addiction three times but still had relapses. Although Johan has followed treatment, he still has frequent relapses which affects his work ability negatively.

For five years, Johan has been on sick leave from time to time because of his alcohol dependence. Two and a half years ago, he left his job. Since then, Johan has tested in collaboration with the Employment Agency and the Social Insurance Office several jobs, but he has not been able to work more than nine hours a week without experiencing an increase in symptoms related to their alcohol dependence. This past year, Johan has been on sick leave 100 percent.

NOTE: The patient’s name [Johan or Johanna] and diagnosis [depression, alcohol dependence or lumbago with sciatica] varied between the vignettes. To increase external validity, some context needed to be provided for each diagnosis. But overall, the content of the vignettes remained constant.

**Supplementary File 2. Interaction effect of vignette diagnosis and gender on the odds of a physician thinking 'should get a DP'**

|  |  | **Crude Model (n=1,308)** | **Adjusted Model**  **(n=1,255)** |
| --- | --- | --- | --- |
|  | **Variable** | **OR (CI)** | **OR (CI)** |
| Should get a DP | Vignette diagnosis |  |  |
|  | Low back pain | 1 | 1 |
|  | Depression | 1.74 (1.17 to 2.59) | 1.76 (1.17 to 2.65) |
|  | Alcohol dependence | 0.41 (0.28 to 0.61) | 0.39 (0.26 to 0.58) |
|  | Vignette gender |  |  |
|  | Male | 1 | 1 |
|  | Female | 0.81 (0.55 to 1.19) | 0.81 (0.55 to 1.20) |
|  |  |  |  |
|  | Vignette diagnosis#Vignette gender |  |  |
|  | Low back pain#Female | 1 | 1 |
|  | Depression#Female | 1.18 (0.67 to 2.07) | 1.20 (0.67 to 2.57) |
|  | Alcohol dependence#Female | 1.21 (0.70 to 2.09) | 1.28 (0.73 to 2.24) |

Abbreviations: DP – Disability pension; OR: Odds ratio; CI: Confidence Interval

*Model adjusted for age of physician, gender of physician and experience of physician completing disability pension assessment in past 12 months.

| **Supplementary File 3. Characteristics of the initial study population, respondents and analytic sample** | | | | | | |
| --- | --- | --- | --- | --- | --- | --- |
| **Variable** | **Study population**  **(initial sample)** | | **Respondents** | | **Analytic sample** | |
|  | **n** | **%** | **n** | **%** | **n** | **%** |
| **Total** | 6000 | 100 | 1414 | 24% | 1255 | 20.9 |
| **Gender of physician** |  |  |  |  |  |  |
| Male | 2953 | 49% | 722 | 51% | 650 | 51.2% |
| Female | 3035 | 51% | 691 | 49% | 605 | 48.2% |
| Undefined | 12 | 0% | 1 | 0% | - | - |
| **Location** |  |  |  |  |  |  |
| Major cities | 2642 | 44% | 580 | 41.0% | 521 | 41.5% |
| Regional or rural | 3357 | 56% | 834 | 59.0% | 734 | 58.5% |
| **Age of physician** |  |  |  |  |  |  |
| Average age | 50.97 | - | 52.9 | - | - | - |
| < 35 years | 639 | 11% | 135 | 10% | 114 | 9% |
| 35 to 44 years | 1501 | 25% | 303 | 21% | 278 | 22% |
| 45 to 54 years | 1346 | 22% | 278 | 20% | 246 | 20% |
| 55 to 64 years | 1533 | 26% | 389 | 28% | 344 | 27% |
| 65 to 74 years | 883 | 15% | 277 | 20% | 244 | 19% |
| > 75 years | 96 | 2% | 32 | 2% | 29 | 2% |
| **Type of physician** |  |  |  |  |  |  |
| Psychiatrist | 1783 | 30% | 443 | 31% | - | - |
| GP | 4217 | 70% | 971 | 69% | - | - |
